# Next-generation Sequencing-based genomic profiling: Fostering innovation in cancer care?

**DOI:** 10.6061/clinics/2017(10)01

**Published:** 2017-10

**Authors:** Gustavo S. Fernandes, Daniel F. Marques, Daniel M. Girardi, Maria Ignez F. Braghiroli, Renata A. Coudry, Sibele I. Meireles, Artur Katz, Paulo M. Hoff

**Affiliations:** I Centro de Oncologia, Hospital Sirio Libanes, Sao Paulo, SP, BR; II Instituto do Cancer do Estado de Sao Paulo, Hospital das Clinicas HCFMUSP, Faculdade de Medicina, Universidade de Sao Paulo, Sao Paulo, SP, BR

**Keywords:** Molecular Profiling, Targeted Therapy, Precision, Refractory

## Abstract

**OBJECTIVES:**

With the development of next-generation sequencing (NGS) technologies, DNA sequencing has been increasingly utilized in clinical practice. Our goal was to investigate the impact of genomic evaluation on treatment decisions for heavily pretreated patients with metastatic cancer.

**METHODS:**

We analyzed metastatic cancer patients from a single institution whose cancers had progressed after all available standard-of-care therapies and whose tumors underwent next-generation sequencing analysis. We determined the percentage of patients who received any therapy directed by the test, and its efficacy.

**RESULTS:**

From July 2013 to December 2015, 185 consecutive patients were tested using a commercially available next-generation sequencing-based test, and 157 patients were eligible. Sixty-six patients (42.0%) were female, and 91 (58.0%) were male. The mean age at diagnosis was 52.2 years, and the mean number of pre-test lines of systemic treatment was 2.7. One hundred and seventy-seven patients (95.6%) had at least one identified gene alteration. Twenty-four patients (15.2%) underwent systemic treatment directed by the test result. Of these, one patient had a complete response, four (16.7%) had partial responses, two (8.3%) had stable disease, and 17 (70.8%) had disease progression as the best result. The median progression-free survival time with matched therapy was 1.6 months, and the median overall survival was 10 months.

**CONCLUSION:**

We identified a high prevalence of gene alterations using an next-generation sequencing test. Although some benefit was associated with the matched therapy, most of the patients had disease progression as the best response, indicating the limited biological potential and unclear clinical relevance of this practice.

## INTRODUCTION

The enhancement of molecular biology techniques in the past decades and the subsequent understanding of cell-cycle control mechanisms have helped to define the hallmarks of cancer and initiate the era of targeted therapy 1,2. The development of imatinib for the treatment of patients with chronic myeloid leukemia has led to an impressive improvement in the clinical management of this disease, initiating a race to develop and clinically test small-molecule inhibitors and monoclonal antibodies targeting fundamental effectors involved in cell carcinogenesis 3,4.

Personalized medicine involves matching the right drugs to the right patients. The potential benefit of this approach is very attractive for patients without any remaining conventional therapy available and for whom comprehensive genomic profiling could identify a potential new, targeted therapeutic approach. Next-generation sequencing (NGS) is one of the most advanced technologies applied to deciphering molecular alterations in tumors and enables scientists to rapidly identify numerous mutations in patient tumors 5-7. This growing knowledge has significantly improved pharmaceutical development over the years, leading to some impressive successes in cancer care 1,2.

One of the first studies to demonstrate the potential benefit of matched therapy based on molecular profiling in heavily pretreated patients showed that 98% of the patient tumors had a gene alteration (GA) that could be used as a target. That study reported that 27% of the patients had an increase in progression-free survival (PFS) with treatment based on tumor molecular alterations compared with the PFS obtained with their previous treatment 8. Certain other small, retrospective studies have also provided evidence of a high prevalence of GA in patient tumors and suggested the potential clinical benefit of molecular profiling 9,10; however, the recent prospective and randomized phase II SHIVA trial suggested that targeted agents matched according to GAs outside of their formal indications did not improve PFS 11.

The present retrospective study was conducted to evaluate the role of genomic testing in treatment decisions for patients with heavily pretreated metastatic solid tumors at a single institution.

## METHODS

### Patients

We evaluated consecutive patients with advanced solid and hematological malignancies, whose tumors were subjected to NGS profiling from July 2013 to December 2015 at all oncology units of Hospital Sírio-Libanês in São Paulo and Brasília. Patients 18 years or older who presented with radiological evidence of metastatic disease and failed to respond or progressed on all recognized standard-of-care therapies were eligible for analysis. Patients with non-metastatic solid tumor(s) who did not receive systemic cancer treatment prior to testing or received targeted therapy based on test results considered standard for their disease were excluded. Additionally, outpatients with a loss to follow-up or lack of data concerning treatment after completing the genomic sequencing via NGS were also excluded.

Clinical characteristics, such as age, gender, the location of the primary tumor, performance status based on the Eastern Cooperative Oncology Group (ECOG) score, previous systemic treatment, PFS and overall survival (OS) were obtained from medical records.

We also compared patients who received targeted therapy against ERBB2 (HER2) with patients who received targeted therapy for any other alteration with the exception of HER2.

### Analysis of molecular alterations

Patients with adequate tumor tissue from archival formalin-fixed paraffin-embedded (FFPE) tumor blocks or a minimum of 10 FFPE slides 4 to 5 mm thick were evaluated using commercially targeted NGS assays (FoundationOne and FoundationOne Heme from Foundation Medicine, Massachusetts, USA). This test utilizes the DNA sequencing of 315 cancer-related genes and 28 genes commonly rearranged in cancer. For hematologic malignancies and sarcomas, the FoundationOne Heme test was performed, with DNA sequencing for 405 genes somatically altered in cancer and 31 genes involved in rearrangements in addition to the RNA sequencing of 265 genes. Genomic alterations simultaneously detected by this assay include base substitutions, short insertions and deletions, focal gene amplifications and homozygous deletions (copy number alterations), and select gene fusions and rearrangements 7.

### Treatment

A therapy was considered "matched" if a U.S. Food and Drug Administration (FDA)-approved drug was known to inhibit the functional activity resulting from at least one of the GAs of a patient’s tumor, as indicated in the Foundation Medicine cancer gene panel (FM-CGP) results. The decision whether to use this matched therapy or not was made by the patient’s physician.

### Objectives and statistical analyses

Our primary objective was to evaluate the response rate of the tested patients who received therapy directed by NGS-based genomic profiling. The secondary objectives were to assess the prevalence of genomic and targetable alterations and to determine the PFS and OS obtained with the directed therapies.

All statistical analyses were performed using Predictive Analytics Software (PASW 18) and R Project for Statistical Computing. Tumor response was evaluated through computed tomography imaging and retrospectively assessed using the Response Evaluation Criteria in Solid Tumors (RECIST) version 1.1 12. The PFS and OS data were summarized using the Kaplan-Meier method and compared using the log-rank test. OS was measured from the first day of treatment with the matched therapy until death or the last follow-up. PFS was measured from the first day of treatment until the date of disease progression, death or the last follow-up, whichever came first. All the P-values presented were two-sided, and statistical significance was determined when *p≤*0.05.

This study was approved by the local institutional review board and was conducted in accordance with state and federal regulations.

## RESULTS

### Patient characteristics

From July 2013 to December 2015, 185 patients underwent FM-CGP testing, and 157 patients were eligible for further analysis. Among the 28 patients excluded from the analysis, 19 patients were lost to follow-up or had no data on treatment after the completion of the genomic sequencing test, 6 patients received targeted therapy considered standard for the respective histology, and 3 patients were younger than 18 years old.

The baseline characteristics of the 157 patients available for analysis are described in Table 1. The mean age at diagnosis was 52.2 years (range: 15.3 to 91.2). Sixty-six patients (42.0%) were female, and ninety-one patients (58.0%) were male. Most of the patients had an ECOG performance status of 0 or 1 (84.1%). The mean number of treatment lines was 2.7 (range 1 to 10). The most commonly observed tumor sites were lung (18.5%), colorectal (13.4%) and pancreas (10.8%).

### Genomic test results

Among the 185 consecutive patients whose tumors were analyzed using the FM-CGP, at least one GA was identified in 177 patients (95.6%). The average number of altered genes per patient was 3.9 (standard deviation 3.28). Among those 185 patients, 128 patients (69.2%) had at least one targetable molecular alteration based on an FDA-approved therapy. Most patients (43.7%) had one, while 25.4% had two or more druggable alterations.

### Treatment and response

Regarding the 157 patients eligible for treatment analysis, 24 patients (15.2%) received systemic treatment directed by NGS-based genomic profiling testing. Twelve patients (50%) were male, and twelve patients (50%) were female. The mean age was 46.8 years. The majority of the patients had an ECOG performance of 0 or 1 (90.16%). This population was heavily pre-treated. Most patients (62.6%) had received three or more previous lines of treatment. The most common primary sites were lung (12.5%), colorectal (12.5%) and pancreas (12.5%). The most frequently utilized targeted therapies were everolimus (25.9%), trastuzumab (11.1%) and T-DM1 (7.4%).

Two patients received more than one line of targeted therapy based on the FM-CGP results (one patient received two lines, and the other patient received three lines of different targeted therapies), with the remaining patients receiving only one line of matched therapy (Table 2). Among the patients receiving the 27 different lines of matched therapy, one patient achieved a complete response (CR), and four patients achieved a partial response (PR). Three therapies resulted in stable disease (SD), while the best response to 19 therapies was progression of disease (PD) (Table 2). The overall response rate was 20.8%, and the clinical benefit (CR+PR+SD) was 29.2%.

The patient who had a CR was a female with adenocarcinoma of an unknown primary site who had received three previous lines of systemic therapy. The FM-CGP revealed a HER2 mutation, and the patient was treated with trastuzumab combined with cisplatin and gemcitabine. After five months of treatment, CT revealed a complete radiological response. Four months after the end of the trastuzumab-based therapy, the patient experienced a radiological progression, and T-DM1 was initiated, with SD as the best response.

Among the patients with PRs, two patients had metastatic non-small-cell lung adenocarcinoma (NSCLA) with wild-type EGFR at diagnosis, which progressed after receiving all available standard therapies. The NGS-based genomic profiling results revealed a mutation in EGFR, and a PR was observed with afatinib+cetuximab in one patient and with afatinib alone in the other patient. The third patient also had metastatic NSCLA without an EGFR mutation or ALK translocation at diagnosis, which had progressed despite two different lines of chemotherapy prior to the NGS results. The test results revealed MET/HGF amplification, and this patient experienced a PR with crizotinib. The last patient was a female with metastatic triple-negative breast cancer who had progressed despite eight lines of prior chemotherapy. The NGS test results revealed an NF1 gene mutation, and a PR was observed with everolimus.

**Figure 1:**
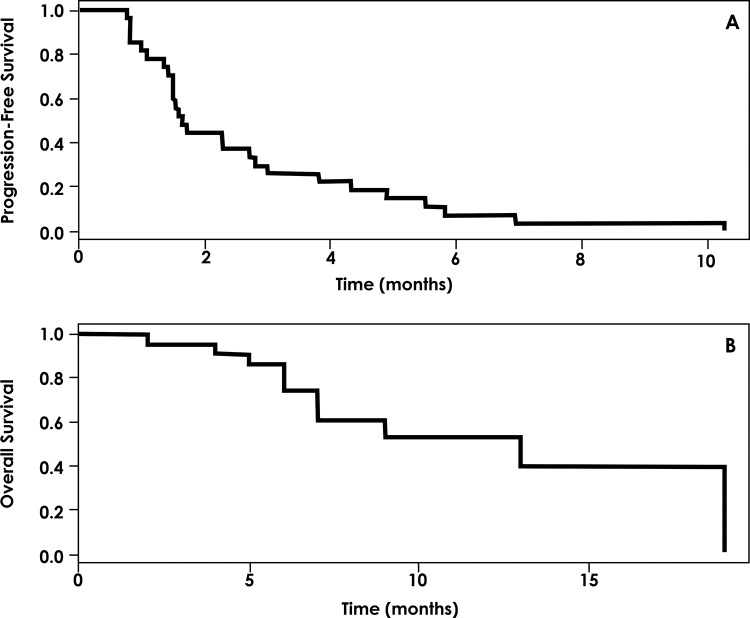
A = Progression-free survival for patients with matched therapy based on the results of the FM-CGP. B = Overall survival for patients with matched therapy based on the results of the FM-CGP.

The median PFS for patients exposed to matched therapy was 1.6 months (range, 0.77-10.3 months), and the median OS was 10 months (range, 0.5-19 months) (Figure 1).

**Figure 2:**
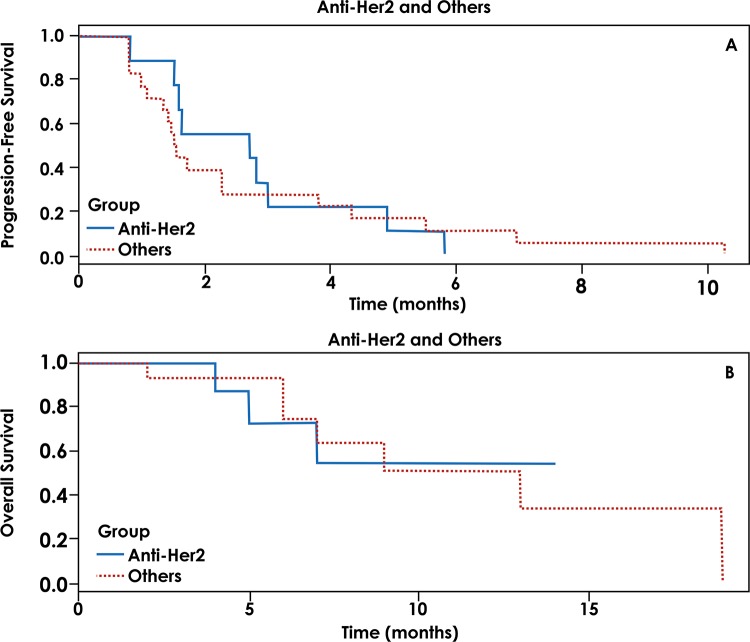
A = Progression-free survival for patients with targeted therapy against HER2 versus any other targeted therapy. B = Overall survival for patients with targeted therapy against HER2 versus any other targeted therapy.

We also evaluated the role of targeted therapy directed against HER2 alterations and performed a comparison between these patients and the remaining patients treated with a matched therapy. A total of 6 patients were treated with a matched therapy against HER2 alterations, and 19 patients were treated with therapies directed at other targets. The median PFS was 2 months for patients who underwent anti-HER2 therapy and 1.5 months for patients who underwent other targeted therapies (*p*=0.332). The OS for patients with anti-HER2 treatment was not reached, and the OS for those treated with non-HER2 targeted therapies was 9 months (*p*=0.866) (Figure 2).

## DISCUSSION

In this cohort of heavily pretreated patients, we observed a high prevalence of at least one targetable alteration in tumor samples (95.6%) with an average of 3.9 mutated genes per patient. The majority of these molecular alterations (approximately 70%) were targetable with FDA-approved therapies. Despite the high prevalence of druggable alterations, only 15% of the patients received a targeted therapy guided by a specific mutation. Although it was not completely clear with all patients, the reasons for not being treated involved patient refusal, treatment cost and no insurance coverage. The outcome analysis revealed a CR in one patient, a PR in four patients and SD in two patients. The survival analysis in these patients was not encouraging with a median PFS of 1.6 months and an OS of 10 months. However, this population was heavily pretreated and had no standard treatment available otherwise.

The prevalence of molecular targets and the response rates observed in our study are consistent with those of other non-randomized trials. A retrospective study of patients with cancer of unknown primary site (CUPS), using the same cancer genome panel as that in our study, found that 96% of the total cases of CUPS harbored at least one alteration, and one patient treated with a matched therapy achieved a CR 10. Similarly, the results of another study indicated that in patients with at least one targetable alteration, a matched therapy compared with treatment without matching was associated with a higher objective response rate (12% *vs*. 5%; *p*<0.0001) and a significantly longer PFS (median, 3.9 *vs*. 2.2 months; *p*=0.001) and survival (median 11.4 *vs*. 8.6 months; *p*=0.04) 9.

Only a few prospective studies are available in the literature with conflicting results. A prospective trial presented at the 2015 American Society of Clinical Oncology (ASCO) meeting compared the outcomes of patients with advanced malignancies who were on a molecular alteration-matched therapy with those on a non-matched therapy. In total, 95% of 339 tested patients had at least one GA indicated by NGS. Patients treated with a matched therapy had significantly improved PFS (median, 3.9 *vs*. 3.3 months; *p*=0.002) and OS (median, 10.8 *vs*. 7.5 months; *p*=0.013) 13. A recent multi-center, open-label, randomized, controlled phase 2 trial of molecularly targeted therapy based on tumor molecular profiling versus treatment based on the physician’s choice in patients with refractory cancer (the SHIVA trial) revealed no difference in PFS between the two arms (median, 2.3 *vs*. 2.0 months, hazard ratio: 0.88, 95% CI 0.65-1.19, *p*=0.41). The objective responses also did not differ between the two groups (4.1% *vs*. 3.4%, *p*=0.19). In a subgroup analysis of patients with alterations in the RAF/MEK pathway, the HR was 0.58. However, this value was not significant, possibly due to the small number of patients in the subgroup 11.

The most interesting result in our series was a patient with an adenocarcinoma of unknown primary site with a HER2 mutation who experienced a CR with trastuzumab-based therapy. Based on this observation, we compared targeted therapies against a HER2 alteration with other therapies. Although clearly exploratory, our data failed to demonstrate a significant difference between targeted therapies directed against HER2 versus those directed at other targets.

Comprehensive genomic profiling has the potential to significantly change clinical practice by individualizing patient treatment. For more than two decades, molecular alterations in tumor cells have been guiding anticancer drug development with increasing success 14,15. The recent advent of NGS technologies has revolutionized the field of human genetics, enabling the fast and cost-effective generation of data 16,17. These technologies have potential applications for many purposes, such as the identification of multiple GAs that can be targeted in personalized therapy 18.

However, despite the latest progress in this technology, our study suggests that only a few patients experienced clinical benefit from targeted therapy based solely on NGS results. Therefore, many points must be considered regarding the clinical use of these data. First, as shown in our study, most tumor samples have more than one targetable GA, and the prioritization of the choices for targeted therapy taking into account the different mechanisms of resistance remains a challenging task 11,19. Second, it is important to emphasize that not all alterations of the genes involved in carcinogenesis will act as “drivers” with the potential to respond to targeted therapy. In addition, many of the rare oncogene variants are of uncertain functional and clinical significance and require further studies 19. The costs associated with NGS and the difficulty in estimating cost effectiveness are also complicating factors.

The evolution of genomics knowledge has consistently demonstrated that solid cancers have extensive heterogeneity among individual tumors and among different regions of the same tumor, which could contribute to treatment failure and drug resistance 20-22. Therefore, as Marco Gerlinger has stated, "reconstructing tumor clonal architectures and the identification of common mutations located in the trunk of the phylogenetic tree may contribute to more robust biomarkers and therapeutic approaches" 20.

Matched therapy appears to benefit a few patients whose cancers have progressed despite all available standard therapies, and how to identify these patients remains elusive. Furthermore, as the development of new drugs progresses, new challenges emerge. In the modern era, immunotherapy has increasingly demonstrated activity in many tissues; therefore, we evaluated the mutational load identified by cancer gene panels, such as the FM-CGP, and the clinical benefit of a PD-1 blockade in non-small-cell lung cancer patients. Patients harboring a high mutational load (more than 7 mutations found in the FM-CGP) had a statistically significant benefit from anti-PD-1 treatment compared with the patients with a low mutational load (median PFS of 14.5 *vs*. 3.4 months, HR: 0.265, *p*=0.005), suggesting that mutational load could be used as a predictive clinical marker for immunotherapy 23.

The present study is limited by its retrospective nature, the small number of patients included, and a possible patient selection bias. However, our study reflects the outcomes of a new approach in oncology practice in a distinct selective population of advanced, heavily treated patients. Our data are similar to those of other retrospective and non-randomized trials that showed some response in certain individuals when targeted therapies were used based on the results of the genomic sequencing of tumor samples; however, the real benefit remains unclear and is probably smaller than anticipated.

Future and ongoing trials will add evidence regarding this matter. The large phase II National Cancer Institute - Molecular Analysis for Therapy Choice (NCI-MATCH) trial will screen 5,000 patients for a targeted treatment guided by tumor GAs 24,25. Similarly, the NCI-MPACT (Molecular Profiling-Based Assignment of Cancer Therapy) trial aims to compare the response rate and/or 4-month PFS for treatment with matched therapy guided by molecular aberrations versus treatment with drugs randomly chosen from a complementary set of agents 24. MyPathway is an ongoing phase II trial evaluating four treatment regimens in a population similar to that in our study, i.e., without available standard treatment. The first analysis of this trial was presented at the 2016 Gastrointestinal Cancer Symposium, and 29 out of 129 patients had some response to targeted therapy, with the most promising results observed with anti-HER2 therapies in patients with colorectal, biliary and bladder cancers. The study is designed to accrue up to 500 patients 26. Similarly, the TAPUR (Targeted Agent and Profiling Utilization Registry) study aims to evaluate the role of commercially available anticancer drugs prescribed for the treatment of patients with advanced cancer that has a potentially actionable genomic variant (NCT02693535).

In conclusion, the advancement of cancer genome knowledge associated with the availability of agents that target altered genes has driven us to consider unusual therapeutic approaches in patients with an excellent performance status and without any conventional therapy available. In this setting, large basket trials will provide knowledge of targetable mutations in different cancers. Furthermore, hypermutated tumors could also be suitable candidates for immune checkpoint inhibitors, and this possibility should be evaluated in future clinical trials.

## AUTHOR CONTRIBUTIONS

Fernandes GS conceived the research, interpreted the collected data, reviewed the manuscript and approved the final version of the manuscript. Marques DF wrote the first draft of the manuscript, interpreted the collected data, reviewed the manuscript and approved the final version of the manuscript. Girardi DM collected the data, wrote the first draft of the manuscript, assisted in interpreting the collected data and approved the final version of the manuscript. Braghiroli MI interpreted the collected data, reviewed the manuscript and approved the final version of the manuscript. Coudry RA Interpreted the collected data, reviewed the manuscript and approved the final version of the manuscript. Meireles SI interpreted the collected data, reviewed the manuscript and approved the final version of the manuscript. Katz A reviewed the manuscript and approved the final version of the manuscript. Hoff PM interpreted the collected data, reviewed the manuscript and approved the final version of the manuscript.

## Figures and Tables

**Table 1:** Characteristics of the population available for analysis (N= 157).

Characteristic	Number (%)
Women	66 (42.0)
Men	91 (58.0)
ECOG	
0	51 (32.5)
1	81 (51.6)
2	16 (10.2)
3	1 (0.6)
Uninformed	8 (5.1)
Mean Age (years)	52.23 years (range 15.3 to 91.25)
Mean prior lines of treatment	2.72 (range 1 to 10)
Tumor Origin	
Lung	29 (18.5)
Colorectal	21 (13.4)
Pancreas	17 (10.8)
Soft tissue	11 (7.0)
Stomach	9 (5.7)
Breast	9 (5.7)
Central nervous system	5 (3.2)
Esophagus	5 (3.2)
Liver	5 (3.2)
Unknown primary site	4 (2.5)
Ovary	4 (2.5)
Adrenal	3 (1.9)
Head and neck	3 (1.9)
Gastric-esophageal junction	3 (1.9)
Uterus	3 (1.9)
Others	26 (16.6)

**Table 2:** Patients treated with targeted therapy based on the results of FoundationOne (N=24).

	First-line Treatment		Second-line Treatment		Third-line Treatment	
Primary site	Treatment	Best Disease Response	Treatment	Best Disease Response	Treatment	Best Disease Response
Unknown	Trastuzumab + Cisplatin + Gemcitabine	CR	TDM-1	SD		
Lung	Cetuximab + Afatinib	PR				
Lung	Afatinib	PR				
Lung	Crizotinib	PR				
Breast	Everolimus + Exemestane	PR				
Head and Neck	Trastuzumab + Docetaxel	SD				
Colorectal	Cabozantinib + Panitumumab	SD				
Liver	Trastuzumab + Docetaxel	PD				
Soft tissue	Everolimus	PD				
Pancreas	Everolimus + Mitomycin + Cyclophosphamide	PD				
Pancreas	Everolimus	PD				
Uterus	Everolimus	PD				
Ovary	Everolimus	PD				
Colorectal	T-DM1	PD	Trastuzumab + Pertuzumab + Capecitabine	PD	Lapatinib + Trastuzumab	PD
Colorectal	Sorafenib	PD				
Pancreas	Dasatinib	PD				
Stomach	Trastuzumab + Carboplatin + Pemetrexed	PD				
Breast	Everolimus	PD				
Brain	Everolimus	PD				
Liver	Pazopanib	PD				
Prostate	Everolimus	PD				
Leukemia	Nilotinib	PD				
Gastric-esophageal junction	Lapatinib + Paclitaxel	PD				
Pancreas	Palbociclib	PD				

Abbreviations: CR, complete response; SD stable disease; PR, partial response; PD, progression of disease.
